# Successful Resection of Leiomyosarcoma Originating from the Inferior Vena Cava with Common Iliac Artery and Vein Reconstruction: A Case Report

**DOI:** 10.70352/scrj.cr.25-0008

**Published:** 2025-05-29

**Authors:** Takashi Miyata, Koki Furuse, Saki Kuwata, Kaori Maruyama, Yuki Shinden, Shota Motoyama, Yuta Sannomiya, Hozumi Tamezawa, Taigo Nagayama, Hisashi Nishiki, Akifumi Hashimoto, Daisuke Kaida, Koichi Okamoto, Hideto Fujita, Nobuhiko Ueda, Daisuke Sakamoto, Yasuhiro Nagayoshi, Hiroyuki Takamura

**Affiliations:** 1 Department of General and Digestive Surgery, Kanazawa Medical University Hospital, Kahoku-gun, Ishikawa, Japan; 2 Department of Cardiovascular Surgery, Kanazawa Medical University Hospital, Kahoku-gun, Ishikawa, Japan

**Keywords:** common iliac artery, common iliac vein, inferior vena cava, leiomyosarcoma

## Abstract

**INTRODUCTION:**

Leiomyosarcomas originating from the inferior vena cava are extremely rare. Because they have a strong tendency to invade the surrounding vital structures, cure can only be achieved by R0 resection.

**CASE PRESENTATION:**

A 59-year-old Japanese woman was referred to our hospital because an abdominal tumor had been detected on a routine ultrasound examination. Computed tomography revealed a 7.0 × 5.3 cm lesion occluding the inferior vena cava to the confluence of the common iliac vein and creating a mass effect on the adjacent aorta and common iliac artery bifurcation. After an open biopsy had yielded a diagnosis of leiomyosarcoma, radical surgery was planned. The tumor was excised *en bloc* together with the inferior vena cava and abdominal aorta and reconstruction performed using artificial blood vessels. Histopathological examination of the resected specimen confirmed it was a leiomyosarcoma originating from the inferior vena cava and invading the aorta and that the surgical margins were negative.

**CONCLUSIONS:**

This report of a rare case of a leiomyosarcoma originating from the inferior vena cava and invading the aorta emphasizes that this combination of pathologies does not preclude curative surgery. However, more data are needed. Further research on leiomyosarcomas is essential for optimizing management and prognosis.

## Abbreviations

IVC
: inferior vena cava
LMS
: leiomyosarcoma
PTFE
: polytetrafluoroethylene

## INTRODUCTION

LMSs have a poor prognosis. They originate from smooth muscle cells and can occur in various sites, including the retroperitoneum, gastrointestinal, and genitourinary systems, and the uterus, skin, and blood vessels.^1,2)^ LMSs arising from the IVC are rare, with fewer than 400 cases having been reported to date.^3,4)^ Surgical resection of the tumor with negative margins is the only treatment proven to improve survival. However, complete resection is characteristically difficult because most patients are asymptomatic, thus their LMS tends to be large and extensive at the time of diagnosis.^5,6)^

We report here a rare case of LMS of the IVC involving the common iliac artery bifurcation. The patient was successfully treated by combined simultaneous resection and reconstruction of the aortoiliac and iliocaval vessels.

## CASE PRESENTATION

**Fig. 1:**
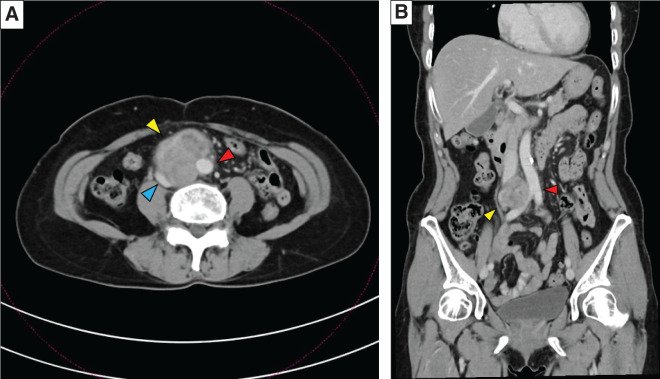
(**A**), (**B**). Abdominal computed tomogram images showed a 7.0 × 5.3 cm multilobulated, heterogeneous, soft tissue mass (yellow arrowhead) in the right hemiabdomen, encasing the inferior vena cava (blue arrowhead) and partially encasing the descending abdominal aorta (red arrowhead).

**Fig. 2:**
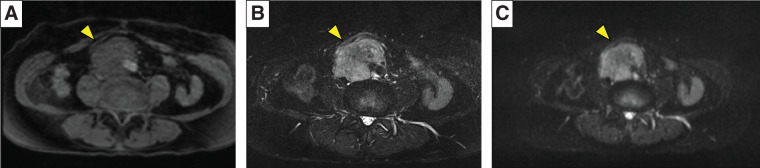
Magnetic resonance images showing a tumor with low intensity on T1-weighted images (**A**, yellow arrowhead), and high intensity on T2-weighted and diffusion-weighted images (**B** and **C**, yellow arrowheads).

**Fig. 3:**
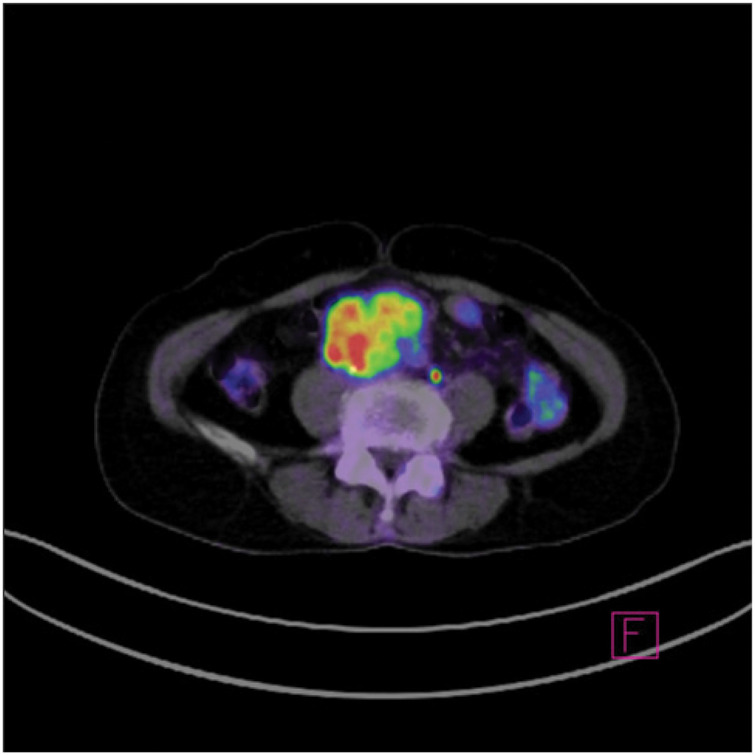
Fluorodeoxyglucose-positron emission tomography image showing increased fluorodeoxyglucose uptake only in the tumor.

A 59-year-old Japanese woman was admitted to our hospital after an abdominal tumor had been diagnosed incidentally by abdominal ultrasound during a medical checkup. Her medical and family histories were unremarkable. She was not taking any medications and did not smoke cigarettes or drink alcohol. On admission, her general condition was unremarkable and physical examination revealed no abnormalities. All laboratory data, including serum titers of the tumor markers carcinoembryonic antigen, cancer antigen 19-9, and cancer antigen 125, were within normal ranges. The portal phase of an abdominal enhanced CT scan revealed an enhanced solitary tumor measuring 7.0 × 5.3 cm occluding the IVC to the confluence of the common iliac vein and contiguous with the lateral border of the aorta and common iliac artery bifurcation (**Fig. 1A**, **1B**, **Supplementary Fig. 1**). There was no pleural or abdominal effusion and no other solid lesions were found in the chest or abdomen. The findings on magnetic resonance imaging were similar to those on CT; namely, there was a mass lesion occupying the IVC from the abdominal aorta to the common iliac artery that had low intensity on T1-weighted images and slightly higher intensity on T2-weighted and diffusion-weighted images (**Fig. 2A**–**2C**). Positron emission tomography showed that the tumor was the only site of increased uptake of 8.95 18F-fluorodeoxyglucose (SUVmax). No distant metastasis was detected (**Fig. 3**). It was difficult to make a definitive preoperative diagnosis, the likelihood being that the tumor was some type of retroperitoneal malignancy, including malignant lymphoma. An open needle biopsy performed to determine the treatment plan yielded a diagnosis of LMS of IVC invading the abdominal aorta. Surgical resection was performed 20 days after the open biopsy with the patient’s consent.

**Fig. 4:**
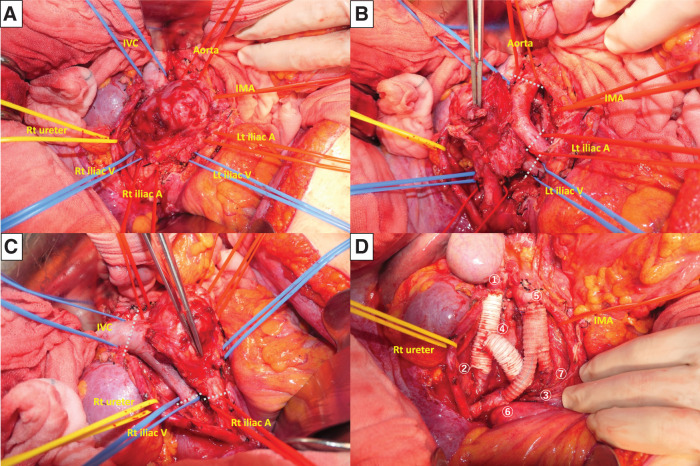
Photographs of operative findings. (**A**) The tumor occupies the lumen of the inferior vena cava, being located within the cranial to the confluence of the left and right iliac veins. (**B**) The tumor has invaded the abdominal aorta and the left common iliac artery; the abdominal aorta and the left common iliac artery and vein were divided along the white lines in the figure. (**C**) Similarly, the tumor has invaded the right common iliac artery; the inferior vena cava and the right common iliac artery and vein were divided along the white lines in the figure. (**D**) Photograph taken after tumor removal and revascularization; reconstruction was performed in the same sequence as the numbers in the photographs. A, artery; IMA, inferior mesenteric artery; IVC, inferior vena cava; Lt, left; Rt, right; V, vein

**Fig. 5:**
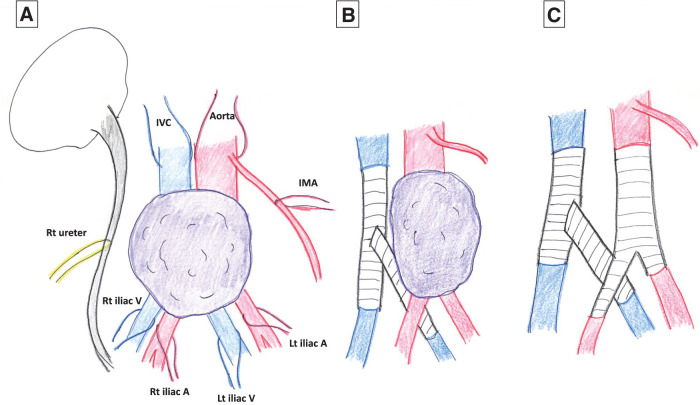
Operative schematic illustrations. (**A**) The specimen was connected only by the arteries and veins. (**B**) Immediately after the iliac vein reconstruction is completed. (**C**) After tumor removal and aortoiliac reconstruction. A, artery; IMA, inferior mesenteric artery; IVC, inferior vena cava; Lt, left; Rt, right; V, vein

After laparotomy, Kocher and right colon mobilization were performed and the small intestine and colon were completely mobilized cranially. The retroperitoneum was incised and the right ureter taped, followed by taping of the left and right common iliac arteries. Next, several lumbar arteries were ligated and transected, and the abdominal aorta was taped cranially to the tumor. During this process, the inferior mesenteric artery was detached from the tumor and preserved. The IVC was then taped cranially to the tumor. Several lumbar veins were also ligated and transected. Finally the left and right common iliac veins were taped. It was found that the tumor had not invaded the vertebral body, enabling ligation and transection of the dorsal cords, which completed the dorsal mobilization of the tumor. Finally, the specimen was connected only by the arteries and veins: the extent of the tumor was as diagnosed preoperatively (**Figs. 4A**–**4C**, **5A**). After intravenous injection of heparin, the IVC and the left and right common iliac arteries were clamped. The IVC and the right common iliac vein were then divided and reconstructed using an artificial blood vessel, a 6 cm Gore-Tex e-PTFE graft (φ20 mm; W. L. Gore & Associates G.K., Tokyo, Japan). The IVC was reconstructed first, followed by the right common iliac vein. The left common iliac vein was then divided and anastomosed to the artificial blood vessel. Next, another graft, also a 6 cm Gore-Tex e-PTFE graft (φ20 mm), was passed from the dorsal side of the artery and side-to-end anastomosed to the midpoint of the previously reconstructed vessel after customizing the length and diameter of the anastomosis graft (**Fig. 5B**). Next, the aorta and the left and right common iliac arteries downstream of the inferior mesenteric artery were clamped, the artery transected, and the specimen extracted. A 14-7 mm Y-graft artificial blood vessel was used for aortoiliac reconstruction (**Figs. 4D**, **5C**). The IVC clamp time was 62 min, the aorta clamp time was 31 min, the total operative time was 366 min, and the intraoperative blood loss was 440 mL.

**Fig. 6:**
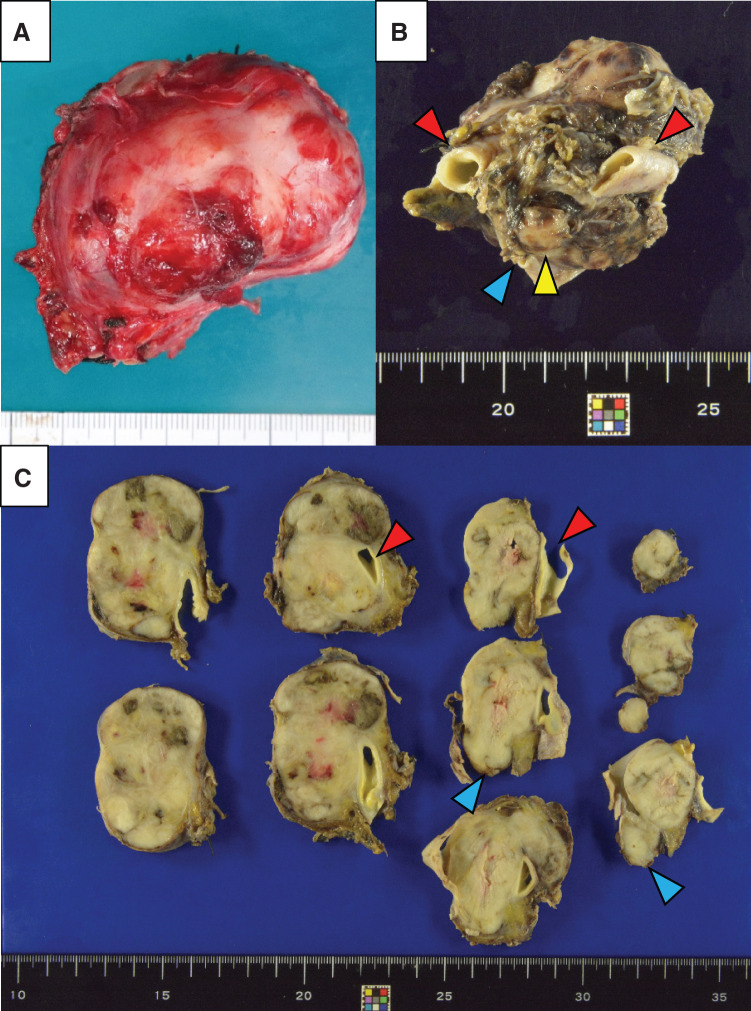
(**A**) Photograph of operative specimen showing a yellowish-white lobulated mass with scattered areas of hemorrhage and necrosis. (**B**), (**C**) The arterial wall is intact (red arrowheads), but the venous lumen contains the tumor and the venous wall is indistinct (blue arrowheads).

**Fig. 7:**
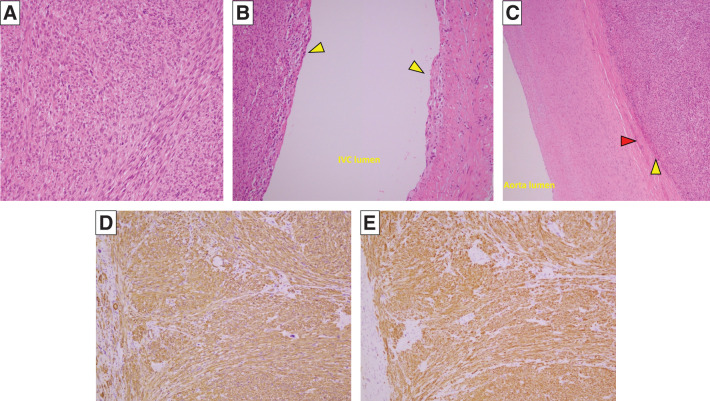
(**A**) Photomicrograph showing that the tumor cells are spindle-shaped, have eosinophilic cytoplasm, and were tangled yet forming bundle-like structures. (**B**) Photomicrograph showing the tumor tissue (yellow arrowheads) is filling the inferior vena cava up to just deep to the endothelium. (**C**) In the artery, the tumor tissue (yellow arrowhead) has invaded the adventitia (red arrowhead), but not the arterial media (**A**–**C**, original magnification, ×100; hematoxylin and eosin stain). The tumor cells were generally (**D**) αSMA-positive and (**E**) desmin-positive. (**D**, **E**, original magnification, ×100). α-SMA, α-smooth muscle actin

Grossly, the resected specimen was a yellowish-white and lobulated nodular tumor measuring 75 × 55 × 52 mm, occupying the lumen of the IVC (**Fig. 6A**–**6C**). Microscopically, the tumor cells were spindle-shaped, had eosinophilic cytoplasm, and were intertwined with fascicle-like structures. Polygonal and multinucleated atypical cells were also identified. The tumor extended to just deep of the endothelium of the veins and to the adventitia in the arteries (**Fig. 7A**–**7C**). Immunostaining showed that the atypical cells were α-smooth muscle actin- and desmin-positive (**Fig. 7D**, **7E**). Therefore, the final diagnosis was an LMS originating from the IVC. The operative time was 366 min and intraoperative blood loss 440 mL. Postoperative antithrombotic therapy comprised heparin for 5 days, followed by oral Bayer aspirin. There was no evidence of graft blood flow ischemia or thrombosis. The patient was discharged 14 days after surgery, having had no complications. There was no CT evidence of tumor recurrence in the subsequent 6 months.

## DISCUSSION

LMS is the second most common primary retroperitoneal tumor in adults.^1,2)^ LMS originating from the IVC, which was first reported in 1871 by Perl and Virchow,^7)^ is an extremely rare, malignant, soft tissue sarcoma, accounting for only 0.5% of all soft tissue sarcomas in adults.^3,4)^ Due to its rarity, poor prognosis, and aggressive nature, its clinical features and optimal means of diagnosis are not well documented.

LMS most often occurs in women, the male–female ratio being 82.6%. The average age at onset is reportedly in the fifties and sixties.^8)^ Because blood tests are often inconclusive, provisional preoperative diagnoses are almost always made by imaging tests such as CT or magnetic resonance imaging; however, these modalities are not sufficient to establish a definitive diagnosis of LMS.^4,6,8)^ It is particularly important to definitively diagnose tumors involving the IVC. The European Society of Medical Oncology recommends diagnosis by needle biopsy guided by CT or ultrasound when a primary leiomyosarcoma of the IVC is suspected. It is recommended that the specimen be obtained intravenously via a catheter.^9)^

Surgery is the first line of treatment for LMS. However, making a diagnosis is difficult because LMS has no specific symptoms and physical exams often fail to reveal any abnormalities, even in the advanced stages of the disease.^8,9)^ Further complicating treatment is the high likelihood that LMS of the IVC will require a combined IVC resection because the standard of care is complete *en bloc* resection of the tumor along with the involved surrounding structures.^10–12)^ Previously, caval invasion was considered as evidence of locally advanced disease and therefore a contraindication to surgery. However, improvements in vascular surgery techniques have made the required procedures a feasible means of treating these cases.^13)^ Resecting the IVC necessitates reconstruction with a banked venous homograft or PTFE graft if a total venous resection is required, whereas ligation alone may be possible if there is a patent collateral venous network.^14)^

This case report highlights the important fact that an acceptable prognosis can be expected with an aggressive surgical approach incorporating simultaneous aortoiliac and iliocaval reconstruction. To the best of our knowledge, no reports of patients undergoing simultaneous resection and reconstruction of the aortoiliac artery and iliac vena cava bifurcation for LMS have been published in English. In the present case, we considered that, given the length of the missing segment and the need for enough strength to resist compression in the abdomen, it would be prudent to strengthen support for the iliocaval reconstruction by directly customizing the length and anastomosis of the iliac vein reconstruction, for which purpose we created the confluence using a ringed, reinforced e-PTFE graft, which reportedly provides the best results,^15)^ instead of a Y-graft artificial blood vessel. The Gore-Tex e-PTFE graft does not have a Y-shape, and separate reconstruction is required. In addition, the left common iliac vein had to be replaced with an artificial vessel due to tumor invasion and the length had to be adjusted intracorporeally and anastomosed to the IVC. Therefore, we chose to perform venous reconstruction first, since it would be difficult to anastomose the IVC and the left common iliac vein if the abdominal aorta was reconstructed first. Iliocaval reconstruction was completed first, followed by the aortoiliac reconstruction. Our procedure achieved complete resection with clear surgical margins in safety.

Optimal treatment strategies for LMS have not been established because there are too few available data regarding the use of chemotherapy, radiation therapy, or both in combination with surgical resection.^16)^ Thus, there is still a need to examine evidence for the effectiveness of treatments in improving the prognosis of LMS. Of note, it has been established that achieving a negative margin is essential for the definitive cure of LMS. The impact of clear surgical margins has been directly demonstrated by 2 case series of LMS. In these, Hines et al.^17)^ and Hollenbeck et al.^18)^ reported that they achieved 5-year survival rates of 68% and 33%, respectively, in patients with negative margins, whereas no patient with positive margins survived for 5 years in either series. Although primary LMS of the IVC typically has a long asymptomatic period and is often at an advanced stage by the time of diagnosis, we propose that surgical treatment should still be considered for these patients, even if simultaneous resection of the IVC and aorta is required, because it is possible to achieve long-term survival.

## CONCLUSIONS

In conclusion, evidence for the use of radiation, chemotherapy, and chemoradiotherapy for LMS is still contentious, the current best treatment being *en bloc* resection with clear surgical margins. Therefore, we suggest that a surgical team consisting of gastroenterological and cardiovascular surgeons be assembled to address the oncological and vascular complexities in patients with LMS with vascular invasion. Using such a team enabled us to successfully perform a combined resection and reconstruction of the iliac arteries and veins in a patient with the rare condition of LMS originating from the IVC and invading the aorta.

## SUPPLEMENTARY MATERIALS

Supplementary Figure Preoperative abdominal contrast CT scan shows the location of the tumor occupying the IVC lumen and invading the aorta.
IVC, inferior vena cava

## ACKNOWLEDGMENTS

We thank Dr Trish Reynolds, MBBS, FRACP, from Edanz (https://jp.edanz.com/ac) for editing a draft of this manuscript.

## DECLARATIONS

### Funding

No authors have direct or indirect commercial and financial incentives associated with publishing the article.

### Authors’ contributions

T.M, D.S., Y.N., and H. Takamura performed the surgery, contributed to the discussion, and were responsible for the patient’s perioperative management.

T.M drafted the manuscript.

K.F., S.K., K.M., Y. Shinden, S.M., Y. Sannomiya, H. Tamezawa, T.N., H.N., A.H., D.K., K.O., H.F., and N.U. also contributed to the discussion.

All authors have approved the manuscript.

### Availability of data and materials

Availability of data and materials data sharing is not applicable to this article, as no datasets were generated or analyzed during the current study.

### Ethics approval and consent to participate

This report has been performed in accordance with the Declaration of Helsinki, and this work does not require ethical considerations or approval.

### Consent for publication

Written informed consent was obtained from the patient for publication of this case report and any accompanying images. A copy of the written consent is available for review by the Editor-in-Chief of *Surgical Case Reports*.

### Competing interests

The authors declare that they have no competing interests.
