# Supplementary material for: Successful Resection of Leiomyosarcoma Originating from the Inferior Vena Cava with Common Iliac Artery and Vein Reconstruction: A Case Report
Source: Surg Case Rep. 2025 May 29;11(1):25-0008. doi: 10.70352/scrj.cr.25-0008 (PMC12129700; doi:10.70352/scrj.cr.25-0008)

Fig.1 Supplementary Figure

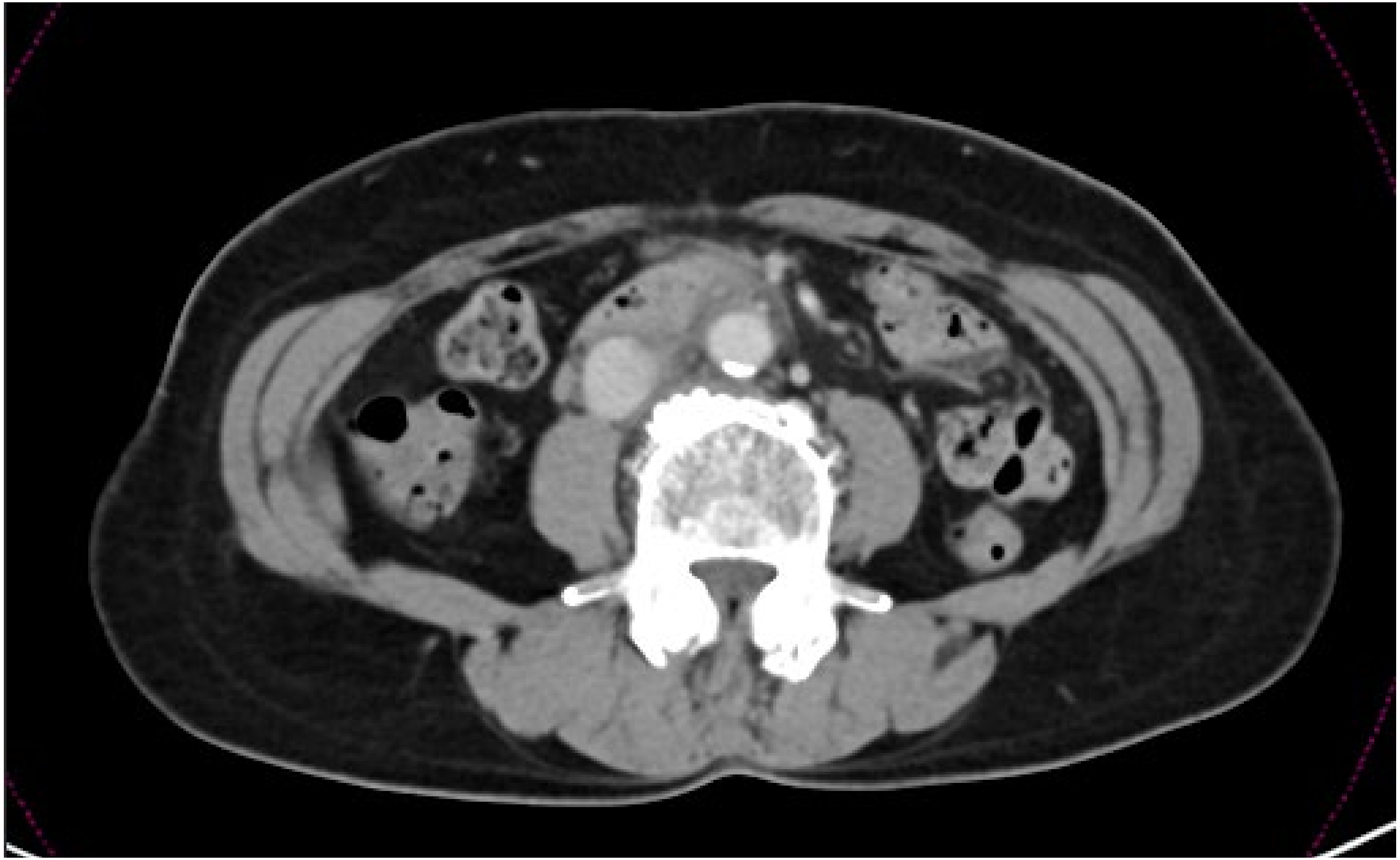

Fig.1 Supplementary Figure

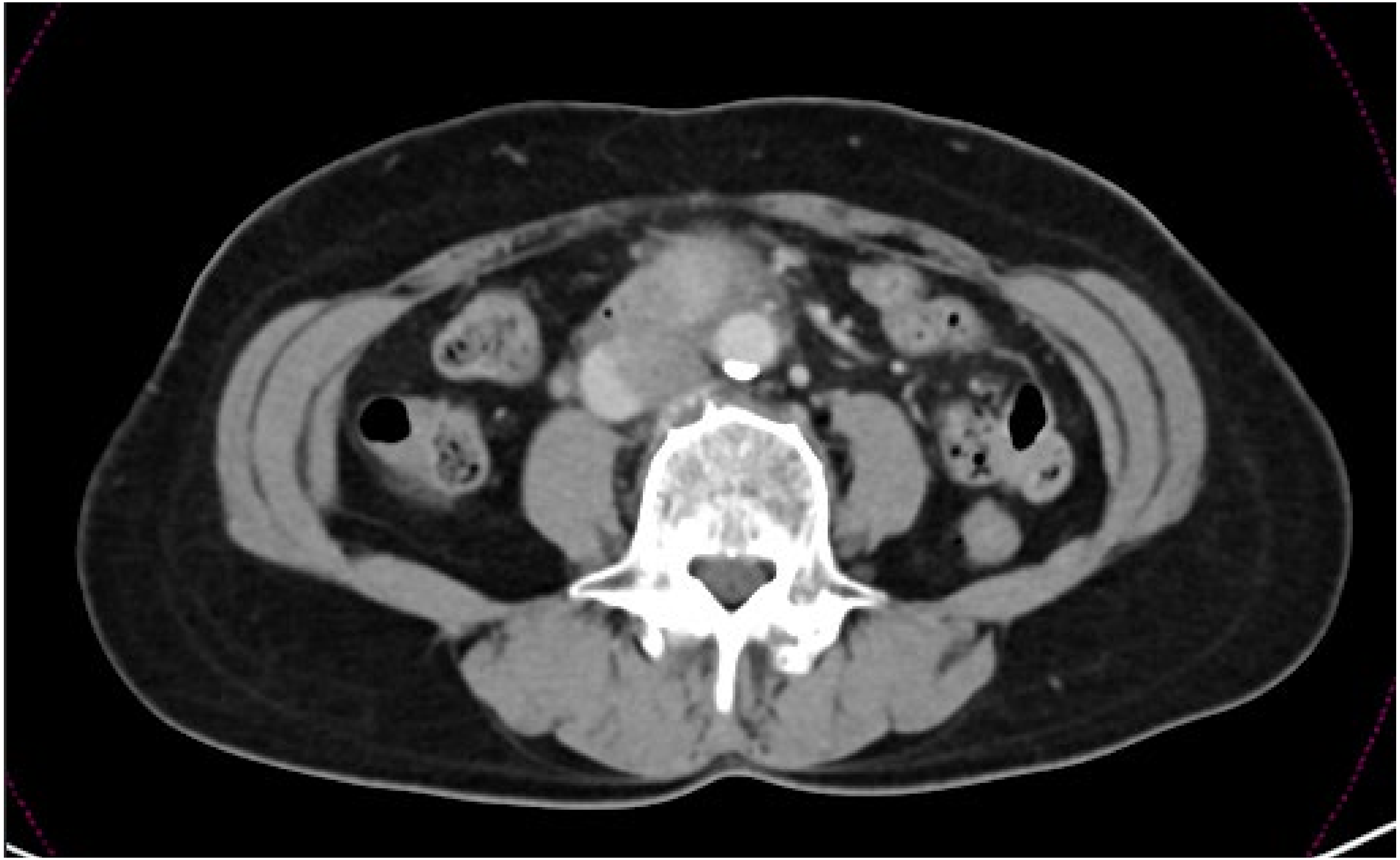

Fig.1 Supplementary Figure

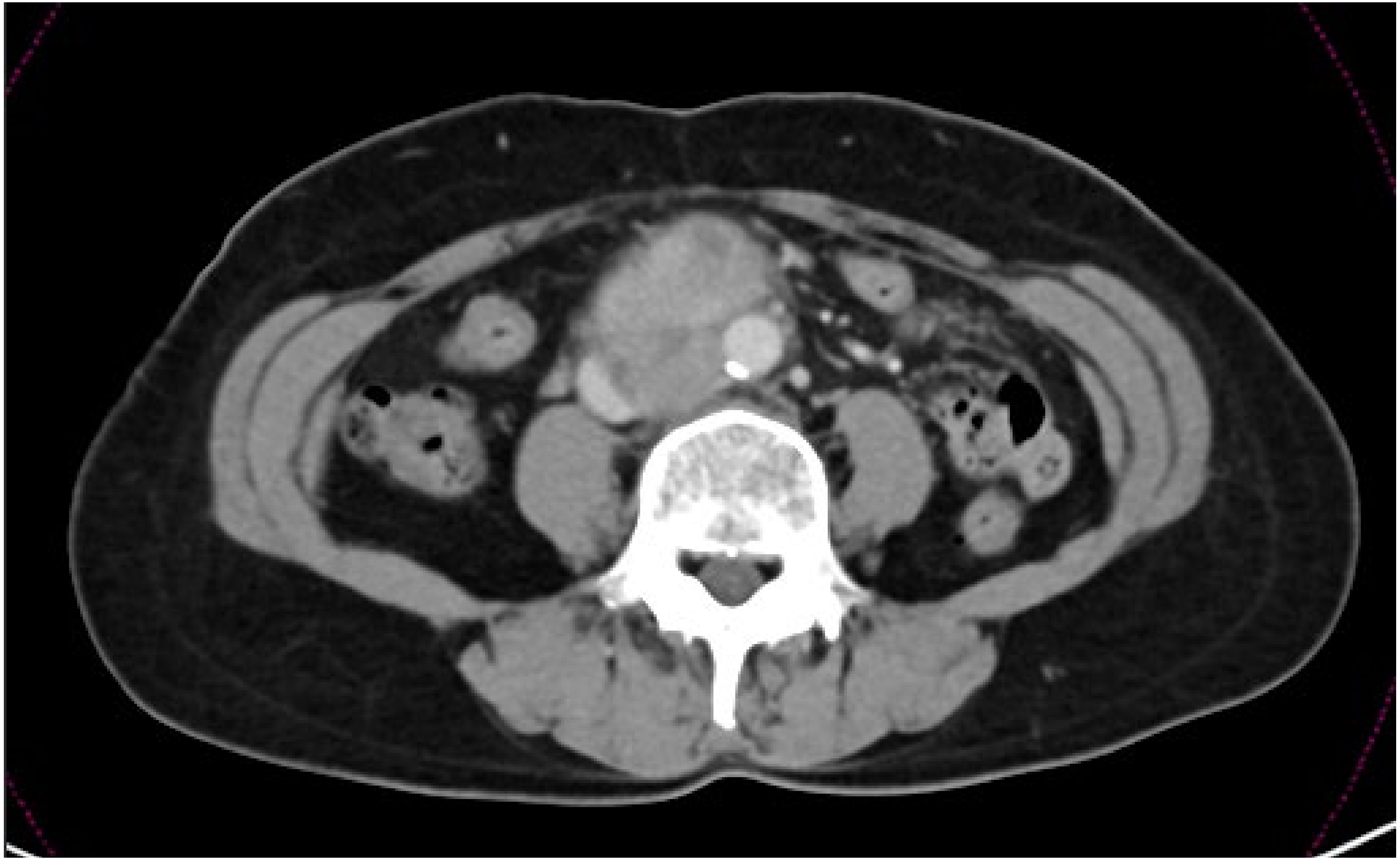

Fig.1 Supplementary Figure

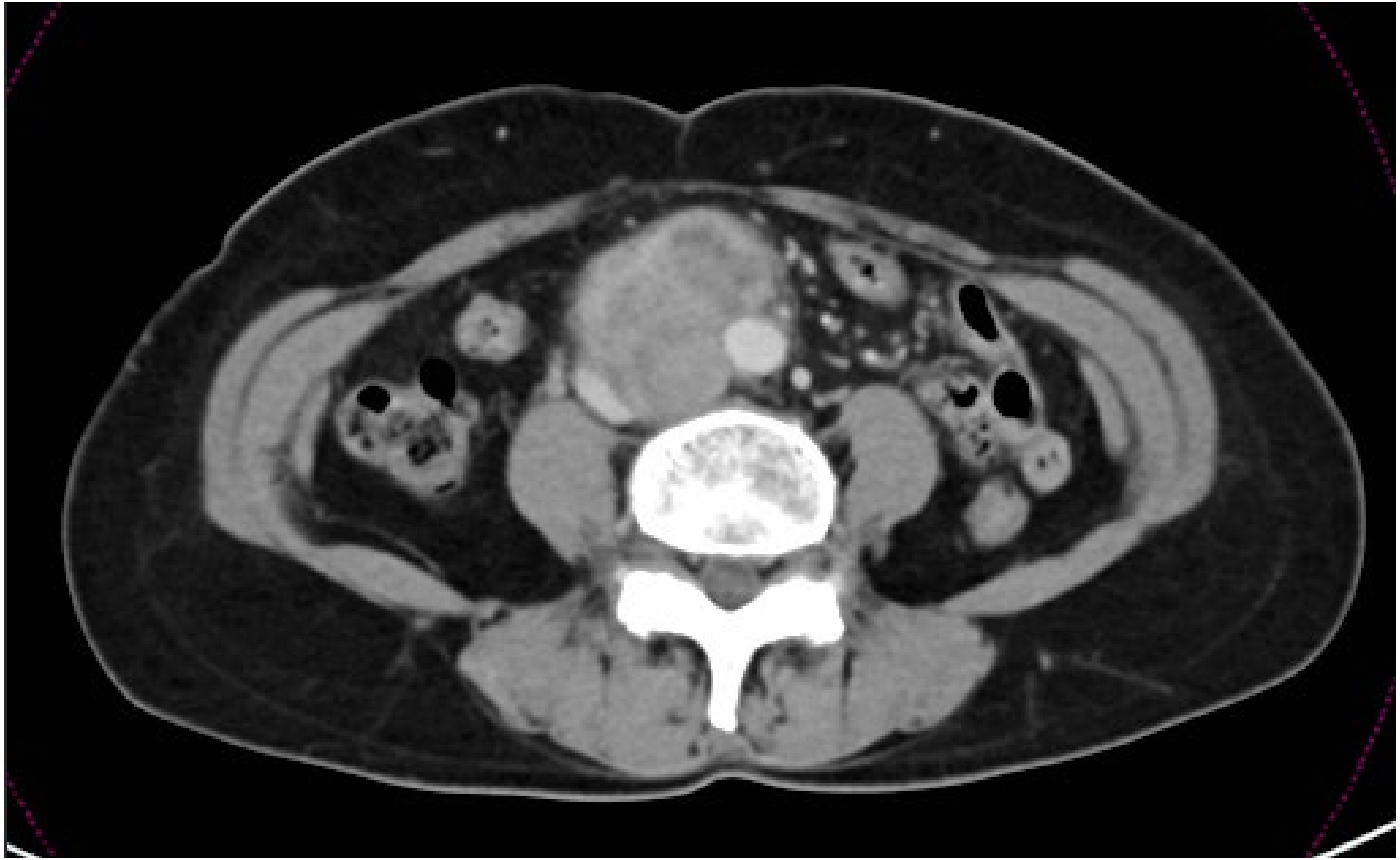

Fig.1 Supplementary Figure

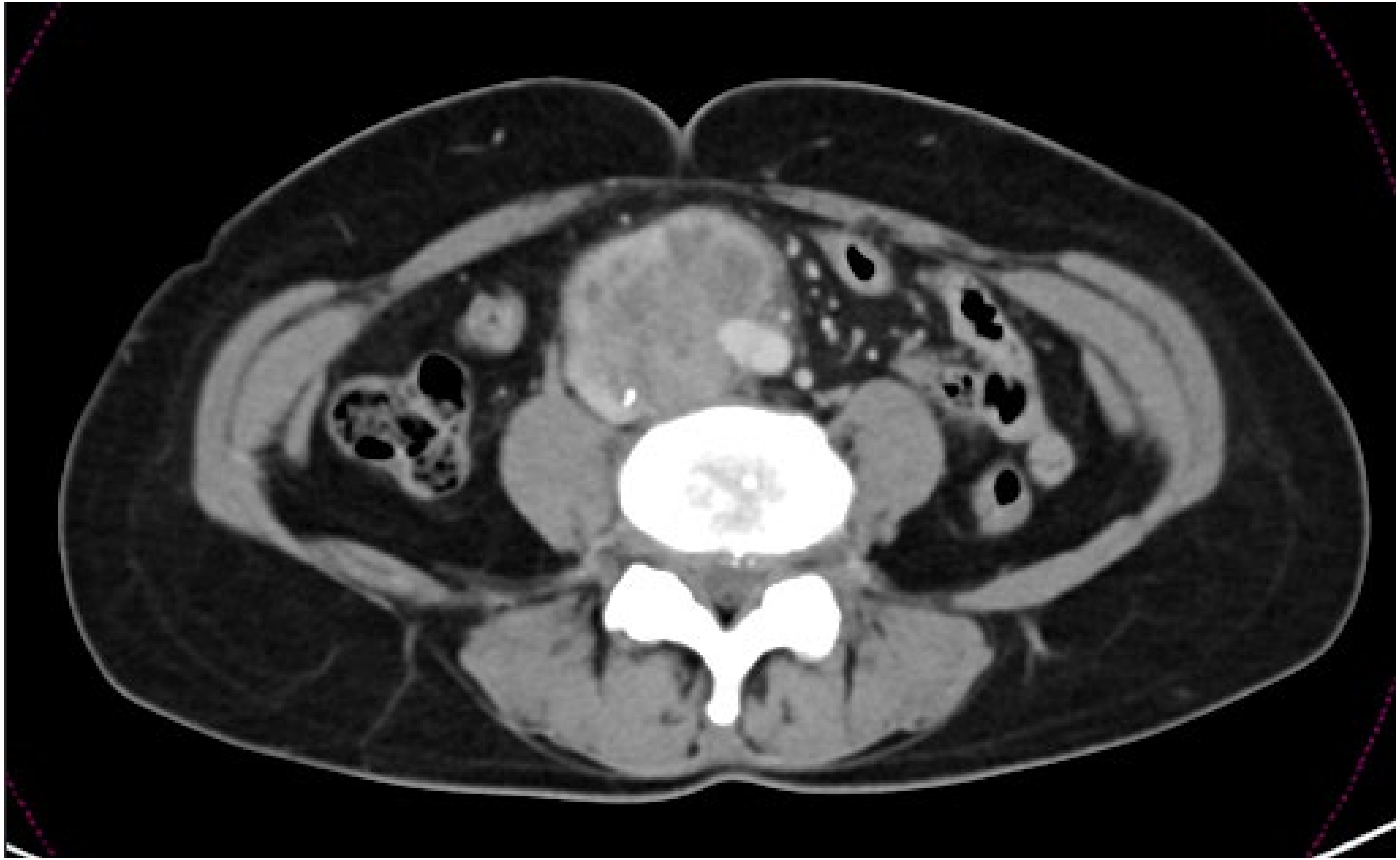

Fig.1 Supplementary Figure

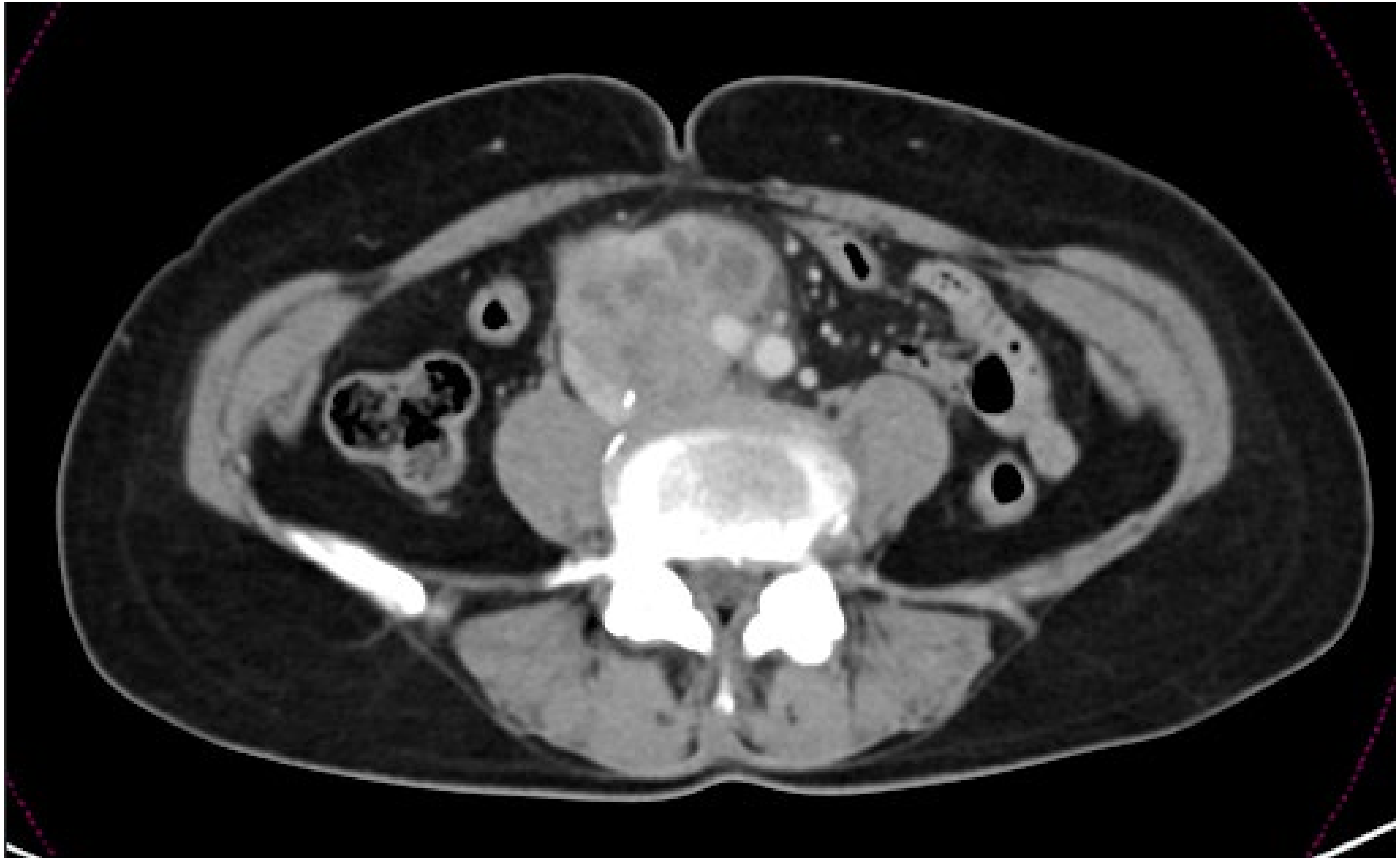

Fig.1 Supplementary Figure

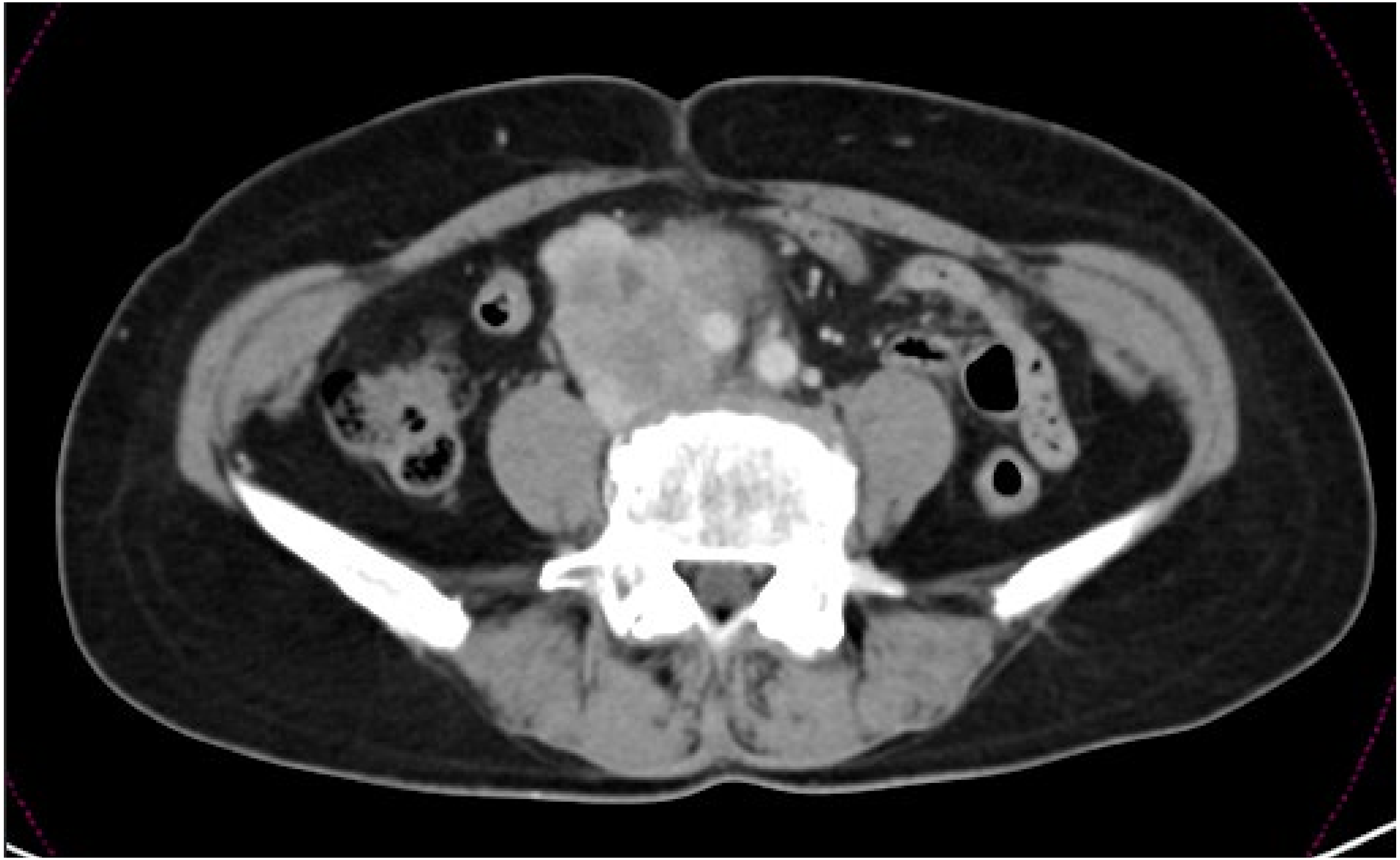

Fig.1 Supplementary Figure

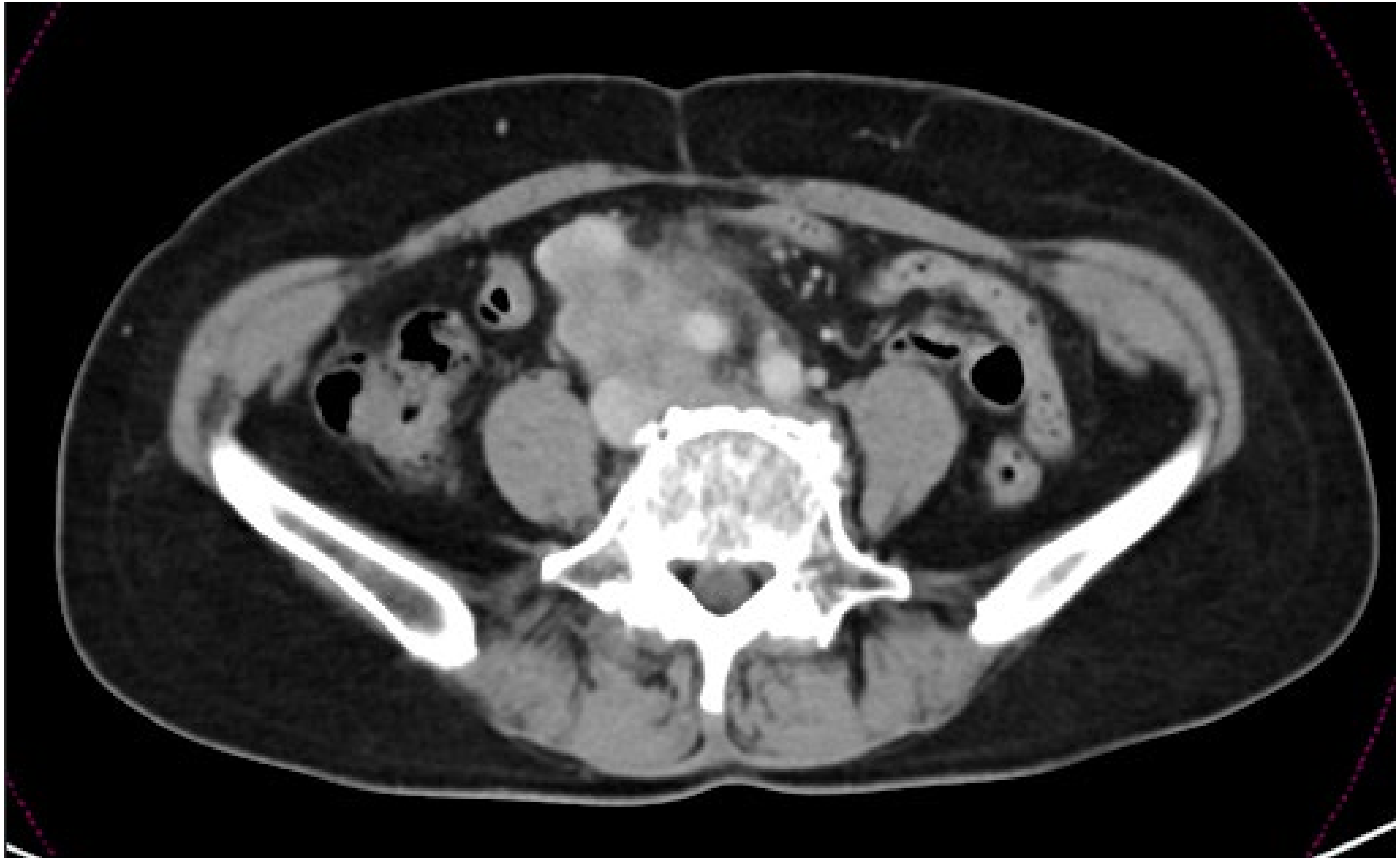

Fig.1 Supplementary Figure

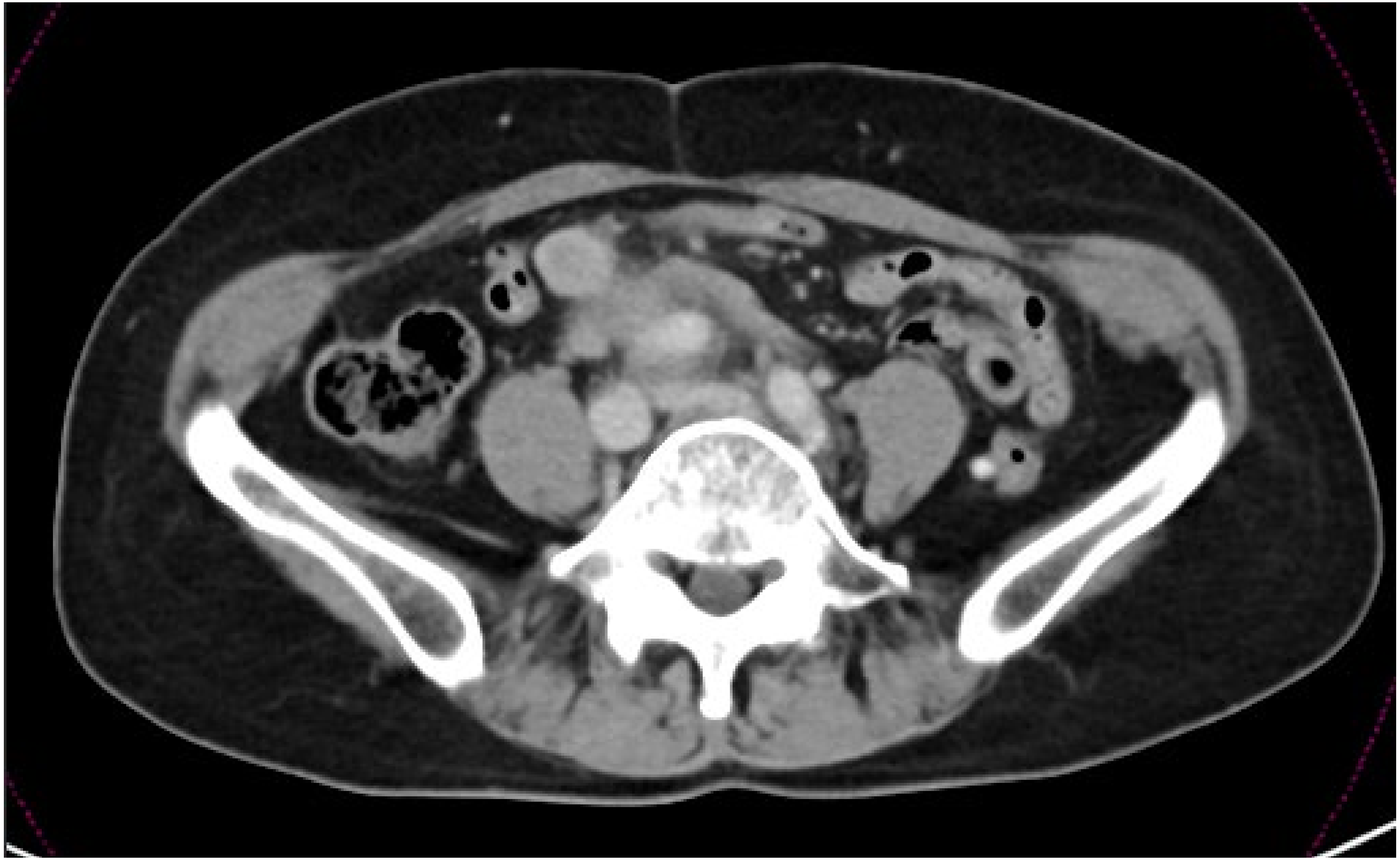

Supplement: Supplementary Figure — Preoperative abdominal contrast CT scan shows the location of the tumor occupying the IVC lumen and invading the aorta. IVC, inferior vena cava [file scr-11-01-25-0008-s001.pdf]
